# Diet, Physical Exercise, and Gut Microbiota Modulation in Metabolic Syndrome: A Narrative Review

**DOI:** 10.3390/life16010098

**Published:** 2026-01-10

**Authors:** Ana Onu, Andrei Tutu, Daniela-Marilena Trofin, Ilie Onu, Anca-Irina Galaction, Cristiana Amalia Onita, Daniel-Andrei Iordan, Daniela-Viorelia Matei

**Affiliations:** 1 Doctoral School, Grigore T. Popa University of Medicine and Pharmacy Iasi, 700115 Iasi, Romania; chirila.ana@d.umfiasi.ro (A.O.); andrei.tutu@umfiasi.ro (A.T.); dr.danielatrofin@gmail.com (D.-M.T.); 2 Department of Biomedical Sciences, Grigore T. Popa University of Medicine and Pharmacy Iasi, 700115 Iasi, Romania; daniela.matei@umfiasi.ro; 3 Department of Morpho-Functional Sciences II (Pathophysiology), Center for Obesity BioBehavioral Experimental Research, Grigore T. Popa University of Medicine and Pharmacy Iasi, 700115 Iasi, Romania; cristiana-amalia.onita@umfiasi.ro; 4 Center of Physical Therapy, Rehabilitation and Wellness, “Dunărea de Jos” University of Galati, 800008 Galati, Romania; daniel.iordan@ugal.ro; 5 Department of Individual Sports and Kinetotherapy, Faculty of Physical Education and Sport, “Dunarea de Jos” University of Galati, 800008 Galati, Romania

**Keywords:** metabolic syndrome, Gut microbiota, physical exercise, dietary patterns, mediterranean diet, dash diet, ketogenic diet

## Abstract

Background: Metabolic syndrome (MetS) is a multifactorial condition characterized by insulin resistance, dyslipidemia, hypertension, and central obesity, and is strongly influenced by lifestyle factors. Growing evidence highlights the gut microbiota as a key mediator linking diet and physical exercise to cardiometabolic health. Objective: This narrative review aims to qualitatively synthesize current evidence on the effects of physical exercise and major dietary patterns including the Mediterranean diet (MedDiet), Dietary Approaches to Stop Hypertension (DASH), and ketogenic/very-low-calorie ketogenic diets (KD/VLCKD) on gut microbiota composition and function, and their implications for metabolic health in MetS. Methods: A qualitative narrative synthesis of experimental, observational, and interventional human and animal studies was performed. The reviewed literature examined associations between structured physical exercise or dietary interventions and changes in gut microbiota diversity, key bacterial taxa, microbial metabolites, and cardiometabolic outcomes. Considerable heterogeneity across studies was noted, including differences in populations, intervention duration and intensity, dietary composition, and microbiota assessment methodologies. Results: Across human interventional studies, moderate-intensity physical exercise was most consistently associated with increased gut microbial diversity and enrichment of short-chain fatty acid (SCFA)-producing taxa, contributing to improved insulin sensitivity and reduced inflammation. MedDiet and DASH were generally linked to favorable microbiota profiles, including increased abundance of *Faecalibacterium prausnitzii*, *Akkermansia muciniphila*, and *Bifidobacterium*, alongside reductions in pro-inflammatory metabolites such as lipopolysaccharides and trimethylamine N-oxide. In contrast, KD and VLCKD were associated with rapid weight loss and glycemic improvements but frequently accompanied by reductions in SCFA-producing bacteria, depletion of *Bifidobacterium*, and markers of impaired gut barrier integrity, raising concerns regarding long-term microbiota resilience. Conclusions: Lifestyle-based interventions exert diet- and exercise-specific effects on the gut microbiota–metabolism axis. While MedDiet, DASH, and regular moderate physical activity appear to promote sustainable microbiota-mediated cardiometabolic benefits, ketogenic approaches require careful personalization, limited duration, and medical supervision. These findings support the integration of dietary quality, exercise prescription, and individual microbiota responsiveness into translational lifestyle strategies for MetS prevention and management.

## 1. Introduction

Metabolic syndrome (MetS) represents a cluster of interrelated metabolic and cardiovascular (CV) abnormalities that markedly increase the risk of type 2 diabetes mellitus (DM2), cardiovascular disease (CVD), stroke, and atherosclerosis, constituting a major global public health burden. The concept was first introduced by Reaven in 1988 as “syndrome X,” highlighting insulin resistance, impaired glucose tolerance, dyslipidemia, hypertension, and increased coronary heart disease risk as interrelated pathophysiological components [1].

MetS is not a single disease entity but a constellation of CVD risk factors, defined differently by major health organizations, with the WHO, NCEP ATP III, and IDF criteria being the most widely used in research and clinical practice. Individuals with MetS have a substantially increased risk of developing DM2, with reports indicating up to a five-fold higher risk, and the condition is increasingly prevalent among younger populations due to sedentary behavior, obesity, and poor dietary habits [2,3].

Hypertension in MetS is closely linked to insulin resistance (IR), hyperinsulinemia, and sympathetic nervous system (SNS) overactivity. IR impairs vasodilation, promotes renal sodium retention, and increases circulating free fatty acids, leading to oxidative stress (OS), vasoconstriction, and elevated blood pressure (BP). Obesity plays a central role in MetS pathogenesis by promoting chronic inflammation and OS, resulting in mitochondrial dysfunction, endothelial impairment, reduced nitric oxide (NO) bioavailability, and worsening IR [3,4].

Inflammatory biomarkers are consistently elevated in MetS and closely associated with central obesity, underscoring the role of chronic low-grade inflammation in its pathophysiology. MetS is characterized by a complex interplay between IR, chronic low-grade inflammation, and OS, which together contribute to endothelial dysfunction, adipose tissue dysregulation, and increased cardiometabolic risk. These interconnected mechanisms reinforce each other, creating a self-perpetuating cycle of metabolic deterioration [5,6,7,8,9].

Visceral adipose tissue acts as an active endocrine organ, secreting adipokines such as leptin, adiponectin, and resistin that regulate insulin sensitivity, vascular tone, inflammation, and BP. Dysregulation of these adipokines contributes to metabolic imbalance, endothelial dysfunction, and atherogenesis. Energy homeostasis is further modulated by the autonomic nervous system (ANS) and hypothalamic–pituitary–adrenal (HPA) axis, with chronic stress promoting sympathetic dominance, IR, central obesity, and MetS development [9,10].

The gut microbiota has emerged as a key regulator of metabolic homeostasis through the microbiota–gut–brain axis (MGBA), integrating neural, endocrine, and immune pathways [11]. Dysbiosis is associated with reduced microbial diversity, increased intestinal permeability, endotoxemia, and chronic low-grade inflammation, all of which contribute to the progression of IR and MetS. Metabolic endotoxemia describes low-grade systemic inflammation driven by increased circulating bacterial lipopolysaccharides (LPS). Alterations in microbiota composition influence appetite regulation, glucose and lipid metabolism, and insulin sensitivity via enteroendocrine signaling and vagal pathways. In contrast, microbiota transplantation and probiotic interventions have demonstrated improvements in metabolic and inflammatory outcomes [9,10,11,12].

Gut microbiota composition is shaped by genetics, geography, lifestyle, and diet. High-fat diets promote dysbiosis and adverse cardiometabolic programming, whereas plant-based and fiber-rich diets enhance microbial diversity and promote the growth of beneficial taxa [13,14]. Maternal high-fat diets have been shown to alter offspring microbiota, increasing the Firmicutes-to-Bacteroidetes ratio (FBR) and predisposing them to hypertension, obesity, and IR later in life. Dietary components such as resistant starches and omega-3 polyunsaturated fatty acids further modulate microbiota composition and metabolic outcomes [15].

Overall, MetS emerges as a complex, multisystem disorder driven by interactions between obesity, inflammation, OS, autonomic imbalance, and gut microbiota dysbiosis. Lifestyle-based interventions targeting physical activity, diet, stress regulation, and microbiota modulation represent promising strategies for the prevention and management of MetS [5,6,7,8,9,10,11,12,13,14,15].

Physical activity is an independent modulator of insulin sensitivity and has been shown to directly influence gut microbiota composition, acting synergistically with diet in the prevention and management of metabolic dysfunction [16].

The gut microbiota represents an attractive therapeutic target due to its high plasticity and responsiveness to lifestyle interventions. Unlike genetic factors, microbiota composition and function can be modulated through non-pharmacological strategies such as diet and physical exercise. This modifiability makes microbiota-targeted interventions low-risk, cost-effective, and suitable for preventive approaches. In the context of metabolic syndrome, lifestyle-driven modulation of the gut microbiota offers a promising complement to conventional prevention and treatment strategies [17].

We hypothesize that major dietary patterns discussed in this review—namely the Mediterranean diet (MedDiet), the Dietary Approaches to Stop Hypertension (DASH) diet, and ketogenic dietary approaches—together with regular physical exercise, favorably modulate gut microbiota composition and function, thereby improving insulin sensitivity, lipid metabolism, BP regulation, inflammatory status, and overall cardiometabolic risk in individuals with MetS. We further hypothesize that combined diet–exercise interventions exert synergistic effects on the gut microbiota–metabolism axis, while inter-individual variability in microbiota responsiveness supports the need for personalized, lifestyle-based strategies in MetS prevention and management.

Unlike previous reviews focusing on individual dietary patterns or cardiometabolic health in general, this review provides a comparative synthesis of major dietary approaches—MedDiet, DASH, and ketogenic diets—together with physical exercise, emphasizing their interactive effects on gut microbiota and their specific clinical relevance for MetS.

A narrative review approach was chosen to allow an integrative and comparative synthesis of heterogeneous evidence spanning dietary patterns, physical exercise, gut microbiota, and metabolic syndrome. This methodology is particularly suited to exploring mechanistic insights, clinical implications, and emerging concepts that cannot be fully captured by systematic approaches alone.

## 2. Effects of Physical Exercise on Microbiota

Systematic physical exercise represents a cornerstone intervention for improving the clinical components of MetS, including insulin sensitivity, lipid profile, BP, and body composition. Exercise is defined as structured, planned, and purposeful physical activity that requires energy expenditure. Regular moderate physical exercise has consistently demonstrated beneficial effects on metabolic health and a reduction in MetS-related risk factors [18,19,20]. Importantly, low physical fitness levels are recognized as independent predictors of MetS development and overall mortality. Notably, many exercise-induced metabolic improvements occur independently of changes in fat mass, indicating that the benefits of exercise extend beyond weight loss alone [21].

Gubert et al. (2020) [22] described the MGBA as a key pathway linking environmental factors to neurological health and disease, particularly in neurodegenerative conditions. The authors emphasized that gut microbiota represents a dynamic, experience-dependent ecosystem strongly influenced by exercise, diet, and stress, with dysbiosis increasingly associated with neurodegenerative pathology. These environmental modulators affect both peripheral mechanisms, including metabolic dysfunction, and central processes involving neuronal and glial function. Physical exercise is associated with increased microbial diversity, fiber, and healthy fat-rich diets support favorable microbiota composition, whereas chronic stress promotes dysbiosis and may accelerate neurodegenerative processes [22]. The adaptive nature of the gut microbiota highlights its potential as a therapeutic target for dysbiosis-related disorders.

Hamasaki (2017) [23] showed that exercise influences the gut microbiota in humans and animals, with evidence pointing to increased microbial diversity and changes in microbiota composition. Tai Chi has numerous health benefits, including improving immune function and reducing gut inflammation, and may positively affect gut microbiota through mechanisms such as vagal modulation and mediation of the HPA axis, but no direct studies have explored this link. The optimal intensity, frequency, and duration of exercise for gut microbiota benefits remain unknown [23].

Monda et al. (2017) [24] demonstrated that the human gut hosts a complex microbial community with essential protective, metabolic, and regulatory roles, contributing to barrier integrity, nutrient processing, immune modulation, and inter-organ signaling. Due to these functions, the gut microbiota has been described as an endocrine-like organ. Exercise is a key modulator of microbiota composition, enhancing microbial diversity, improving the FBR, and promoting beneficial bacterial taxa. These adaptations contribute to gut homeostasis, immune competence, and metabolic health, partly by enhancing the production of short-chain fatty acids (SCFAs). SCFAs—primarily acetate, propionate, and butyrate—are microbiota-derived metabolites that play a key role in maintaining intestinal barrier integrity, regulating immune responses, and modulating metabolic function. Maintaining a stable and diverse microbiota is crucial for effective brain–gut axis communication, and exercise-induced microbiota modulation may represent a therapeutic strategy for correcting dysbiosis and preventing metabolic and inflammatory disorders [24].

Sohail et al. (2019) [25], in an extensive review, showed that disturbances of the microbiome are linked to metabolic diseases, and its composition is influenced by genetic and environmental factors such as nutrition and physical activity. The authors searched the relevant literature using MEDLINE and Google Scholar databases and identified 62 studies suggesting that exercise-induced changes in the microbiome positively influence immune pathways, reduce inflammation, and attenuate OS, contributing to improved metabolic health. These microbial changes release neuroendocrine and immunomodulatory factors, which improve tissue metabolism and energy homeostasis [25]. Exercise has also been shown to improve cardiorespiratory fitness and reduce insulin resistance.

Bonomini-Gnutzmann et al. (2022) [26] conducted a systematic review that examined the effects of high-intensity, high-duration aerobic exercise on the gut microbiota in athletes and its implications for sports performance. Thirteen studies highlighted the negative effects of endurance exercise, including increased intestinal permeability, gut discomfort, and changes in gut microbiota such as elevated *Prevotella* levels and reduced microbial diversity. In contrast, seven studies reported positive outcomes such as increased microbial diversity and increased beneficial gut metabolites. Negative effects were more pronounced in athletes compared to non-athletic subjects. Strength training showed less benefit for gut microbiota compared to aerobic exercise. The findings suggest complex and unclear interactions between exercise, personal factors, and environmental influences on gut microbiota [26].

The gut microbiota, often described as a “second genome,” plays a central role in metabolic regulation and overall health. Zhang et al. (2023) [27] highlighted that physical activity and diet significantly shape gut microbiota composition and function, promoting the generation of beneficial metabolites that support metabolic homeostasis. Adequate nutrition combined with moderate-intensity exercise increases microbial diversity, enhances gut immune barrier function, and modulates metabolic signaling pathways, contributing to the prevention and management of DM2, hyperlipidemia, and related metabolic disorders [27]. These lifestyle-based interventions represent cost-effective and sustainable strategies for improving metabolic health at both individual and population levels.

Yun et al. investigated the effects of moderate and high-intensity exercise on gut microbiota and metabolic health. In a 4-week intervention including non-exercising, moderate, and vigorous-intensity groups, moderate exercise increased *Prevotella*, a genus associated with improved carbohydrate metabolism and reduced inflammation, while decreasing potentially pro-inflammatory taxa such as *Veillonella* and *Dorea*. High-intensity exercise promoted beneficial bacteria (*Bacteroides, Butyricimonas, Odoribacter,* and *Alistipes*), known for supporting gut barrier integrity and producing anti-inflammatory SCFA. These intensity-dependent microbial adaptations were associated with reduced risk of metabolic disorders, underscoring the potential value of personalized exercise prescriptions to optimize gut and metabolic health [28].

Quiroga et al. (2020) [29] investigated the impact of a 12-week combined strength and endurance training program on gut microbiota and inflammation in obese children. In this randomized study involving 39 participants, exercise led to reduced plasma glucose levels and improved muscle strength compared with controls. Metagenomic analyses showed a decrease in obesity-associated taxa, particularly *Proteobacteria* and *Gammaproteobacteria*, alongside an increase in beneficial genera such as *Blautia, Dialister,* and *Roseburia*. Metabolomic findings revealed exercise-induced shifts in SCFA, branched-chain amino acids, and sugars, reflecting a healthier microbial profile. Additionally, physical training attenuated activation of the NLRP3 inflammasome, supporting the role of exercise as an effective non-pharmacological strategy to modulate gut microbiota and reduce obesity-related inflammation in children [29].

Silva et al. (2022) [30] evaluated the effects of moderate- and high-intensity exercise on gut microbiota in relation to metabolic disease risk. Following a 4-week intervention, moderate-intensity exercise increased *Prevotella* abundance while reducing *Veillonella* and *Dorea* species. In contrast, vigorous-intensity exercise enhanced beneficial taxa, including *Bacteroides, Butyricimonas, Odoribacter,* and *Alistipes*. These intensity-dependent microbiota shifts were associated with metabolic conditions such as obesity, diabetes, and inflammatory bowel disease, suggesting that exercise-induced modulation of the gut microbiome may contribute to improved metabolic health [30].

A healthy gut is characterized by high microbial diversity and a balanced ratio of beneficial and pathogenic bacteria, supporting neurotransmitter production and intestinal barrier integrity. This equilibrium enables effective gut–brain communication through neural, endocrine, and immune pathways, influencing cognition and behavior. Physical exercise, ketogenic and Mediterranean dietary patterns, and omega-3 supplementation positively modulate this axis, whereas dysbiosis marked by reduced diversity and pathogenic overgrowth impairs gut barrier function and increases intestinal permeability. Dysbiosis promotes inflammation and elevates circulating lipopolysaccharides and cytokines, which are associated with neurodegenerative processes and cognitive decline [21]. Exercise improves gut health by increasing beneficial, SCFA-producing bacteria such as *Roseburia hominis* and *Faecalibacterium prausnitzii*. *Firmicutes* and *Actinobacteria* have been identified as key exercise-responsive phyla, highlighting the role of physical activity in restoring microbiota balance and supporting gut–brain axis function [31,32].

Allen et al. (2018) [33] examined the effects of a 6-week endurance exercise program on gut microbiota composition and metabolic output in lean and obese adults. Thirty-two previously sedentary participants followed a progressively intensified training protocol. Exercise induced significant microbiota alterations that differed according to obesity status. Lean individuals exhibited increased fecal SCFA, associated with upregulation of bacterial genes involved in SCFA synthesis, alongside increased *Faecalibacterium* and *Lachnospira* and reduced *Bacteroides* abundance. In contrast, obese participants showed reduced *Faecalibacterium* and increased *Bacteroides* and *Collinsella*, with limited improvements in SCFA production [33]. These findings highlight obesity-dependent differences in gut microbiota responsiveness to endurance exercise.

Hoffman-Goetz et al. demonstrated that exercise stimulates key antioxidant enzymes, such as catalase and glutathione peroxidase, along with anti-inflammatory cytokines such as IL-10 and antiapoptotic proteins, including Bcl-2, in intestinal lymphocytes. At the same time, exercise reduces TNF-α, IL-17, and proapoptotic proteins (caspase 3 and 7), leading to an overall reduction in intestinal inflammation [34,35].

Table 1 synthesizes current experimental and clinical evidence demonstrating that physical exercise acts as a key modulator of gut microbiota composition and function, with direct relevance for MetS. Across diverse populations and exercise modalities, moderate-intensity physical activity is consistently associated with increased microbial diversity and enrichment of SCFA-producing taxa, alongside reductions in inflammation, OS, and IR. In contrast, high-intensity or prolonged endurance exercise shows more variable effects, occasionally compromising gut barrier integrity. Collectively, these findings highlight exercise intensity and individual metabolic status as critical determinants of microbiota-mediated cardiometabolic benefits.

Physical exercise is a key modulator of gut microbiota with direct relevance to the pathophysiology of MetS. Regular physical activity influences microbial diversity, bioactive metabolite production, and intestinal barrier integrity, thereby attenuating low-grade inflammation, oxidative stress, and insulin resistance. Moderate-intensity exercise is most consistently associated with beneficial microbiota changes, including enrichment of short-chain fatty acid-producing bacteria and reductions in pro-inflammatory taxa, contributing to lower metabolic endotoxemia and improved insulin sensitivity. However, findings across studies are not uniform, largely due to methodological heterogeneity related to exercise protocols, population characteristics, and microbiome assessment techniques. While moderate exercise enhances microbial diversity, prolonged or high-intensity training may adversely affect gut barrier function in certain populations. Overall, human evidence remains largely associative, highlighting the need for standardized methodologies and personalized exercise interventions to clarify causality and long-term microbiota resilience in MetS.

Exercise-induced modulation of the gut microbiota appears to be both time- and dose-dependent. Acute exercise primarily elicits transient shifts in microbial composition and metabolite production, whereas chronic, regular physical activity promotes more stable increases in microbial diversity and enrichment of SCFA-producing taxa. These adaptations enhance SCFA signaling through receptors such as GPR41 and GPR43, contributing to improved gut barrier integrity, reduced metabolic endotoxemia, and attenuation of low-grade systemic inflammation—key mechanisms in MetS pathophysiology. Emerging evidence suggests that sustained microbiota changes generally require at least 6–12 weeks of moderate-intensity exercise, although responsiveness varies according to age, sex, baseline metabolic status, and initial microbiota composition, highlighting important knowledge gaps and the need for personalized exercise prescriptions.

## 3. Dietary Intervention

Health is strongly influenced by diet, which plays a central role in energy balance, tissue regeneration, and metabolic homeostasis. Improving dietary habits by increasing nutrient-dense foods and limiting harmful components contributes significantly to disease prevention [36]. MetS is closely linked to lifestyle factors, and dietary modification is a key strategy for reducing cardiometabolic risk. Current guidelines recommend a 7–10% body weight reduction within 12 months through caloric deficit and physical activity, targeting a body mass index below 25 kg/m^2^ [37].

Caloric restriction involves reducing energy intake without inducing nutrient deficiencies; however, excessive restriction may impair metabolic regulation and promote IR and hypertension [38]. Eating behavior remains a major modifiable determinant of MetS, although no single optimal dietary pattern has been universally defined. Evidence supports the beneficial role of dietary fiber, adequate protein intake, monounsaturated fatty acids (MUFAs), and polyunsaturated fatty acids (PUFAs) in improving insulin sensitivity and MetS components [39].

Micronutrient disturbances, including vitamin D deficiency, are also implicated in MetS pathogenesis and are associated with IR, hypertension, and CVD [40]. Diets rich in unsaturated fats—particularly omega-3 fatty acids—and low in saturated fats are consistently linked to lower MetS prevalence. Among dietary patterns, the MedDiet and the DASH diet are the most consistently supported strategies for MetS prevention and management, emphasizing nutrient-dense foods and balanced macronutrient intake [41].

Macronutrient quality is critical in MetS management. High-glycemic index diets promote IR, whereas low-glycemic, fiber-rich diets improve glycemic control and satiety. Diets enriched in MUFAs further improve lipid profiles and insulin sensitivity, reinforcing recommendations to limit saturated fats, added sugars, and sodium [42].

Overall, sustainable food-based dietary patterns combined with regular moderate physical activity are favored over extreme caloric restriction or isolated nutrient-focused approaches. Diets rich in omega-3 fatty acids and polyphenols may additionally reduce inflammation and OS, underscoring the systemic benefits of nutritional interventions [43].

Taken together, the MedDiet, DASH diet, and ketogenic/very-low-calorie ketogenic diets (KD/VLCKD) exert distinct effects on gut microbiota composition and metabolic health. MedDiet and DASH are consistently associated with increased microbial diversity, enhanced SCFA-production, preserved gut barrier integrity, and favorable long-term safety profiles. In contrast, KD and VLCKD induce rapid metabolic improvements but are frequently accompanied by reduced microbial diversity, depletion of SCFA-producing taxa, and markers of impaired gut barrier function, raising concerns regarding long-term microbiota resilience.

From a clinical perspective, MedDiet and DASH appear most suitable for long-term prevention and management of MetS, whereas KD/VLCKD may be best reserved as short-term, medically supervised interventions in selected patients. This comparative framework highlights the importance of aligning dietary choice with metabolic goals, gut microbiota health, and long-term sustainability.

### 3.1. Microbiome and the Mediterranean Diet

MedDiet, developed by Ancel Keys in the 1960s and recognized by UNESCO as an intangible cultural heritage, is one of the most extensively studied and globally acknowledged dietary patterns. Observational evidence consistently highlights its protective effects against CVD, DM2, MetS, obesity, cancer, and cognitive decline [44].

MedDiet is based on nutrient-dense foods rich in dietary fiber from complex carbohydrates, PUFAs with anti-atherogenic and anti-inflammatory properties, and bioactive compounds such as flavonoids, phytosterols, terpenes, and polyphenols, which contribute to its antioxidant capacity. Its high micronutrient content further supports immune function and the prevention of nutritional deficiencies [39].

Characterized by high intake of extra virgin olive oil, legumes, whole grains, fruits, vegetables, nuts, fish, moderate red wine consumption, and low saturated fat intake, MedDiet provides vitamins and polyphenols that improve lipid metabolism and insulin sensitivity, reducing CV and all-cause mortality [45,46,47,48]. The cardioprotective role of this dietary pattern was first demonstrated in the Seven Countries Study [49] and later confirmed by the Lyon Diet Heart Study, which reported an approximately 70% reduction in mortality among post-myocardial infarction patients following a MedDiet-style [50].

Growing evidence from systematic reviews and meta-analyses indicates that higher adherence to MedDiet is inversely associated with obesity, MetS, and cardiometabolic disorders, with improvements in BP, lipid profile, glycemic control, and central adiposity, and an estimated 23% reduction in all-cause mortality [51,52,53,54]. Observational studies further support these findings, showing that greater MedDiet adherence is associated with lower BP, fasting glucose, triglycerides, waist circumference, and higher HDL-cholesterol, resulting in a reduced likelihood of MetS across both sexes [55,56].

Vázquez-Cuesta et al. study provides valuable insights into the relationship between the MedDiet and the intestinal microbiome, incorporating age and sex classification with functional metagenomics. Individuals with high adherence to this diet showed an increased abundance of health-associated genera like *Paraprevotella* and *Bacteroides*. Olive oil and fiber correlated with bacteria producing SCFAs and the absence of disease, while red meat consumption was linked to less beneficial microorganisms. These findings emphasize the role of specific MedDiet components in promoting gut and overall health [57].

MedDiet has been shown to beneficially modulate gut microbiota by promoting SCFA-producing bacteria, including *Clostridium leptum*, *Eubacterium rectale*, *Bifidobacterium*, *Bacteroides*, and *Faecalibacterium prausnitzii*, while reducing potentially less favorable taxa such as *Firmicutes* and *Blautia*. These microbiota shifts are associated with reduced inflammation and OS, lower malignancy risk, and improved metabolic health [58]. Furthermore, higher adherence to the MedDiet increases the *Prevotella/Bacteroides* ratio, reflecting the positive effects of dietary fiber and resistant starch on gut microbiota composition and overall gut health [59].

Table 2 synthesizes the robust and convergent evidence supporting the Mediterranean diet as a key lifestyle model for microbiota-mediated cardiometabolic protection. Across observational, interventional, and mechanistic studies, adherence to the MedDiet is consistently associated with enrichment of SCFA-producing bacteria (e.g., *Faecalibacterium prausnitzii*, *Eubacterium rectale*, *Bifidobacterium*) and favorable shifts in fiber-responsive taxa, alongside reductions in pro-inflammatory microbial signatures. Rather than acting through isolated microbial pathways, the MedDiet appears to exert its benefits through a coordinated modulation of microbial diversity, fermentative capacity, and host–microbe interactions driven by its high content of fiber, unsaturated fats, and polyphenols. These shared microbiota-related mechanisms parallel clinically meaningful improvements in lipid metabolism, glycemic control, BP, inflammation, and overall metabolic risk, reinforcing the MedDiet as a benchmark dietary pattern for MetS prevention and management.

Although the MedDiet is generally associated with beneficial gut microbiota modulation and cardiometabolic improvements, results are not entirely consistent across studies. Differences in study duration, population characteristics, dietary adherence, and microbiome sequencing methods contribute to this heterogeneity. Short-term interventions often show rapid increases in SCFA-producing taxa, whereas longer studies suggest partial microbiota adaptation, raising questions about long-term persistence. Moreover, most evidence remains observational, limiting causal inference and highlighting the need to identify responder versus non-responder phenotypes through standardized, longitudinal research.

### 3.2. Microbiome and the Dietary Approaches to Stop Hypertension

The DASH diet emphasizes high consumption of fruits, vegetables, whole grains, low-fat dairy products, fish, poultry, and nuts, while limiting red meat, sweets, added sugars, saturated fats, and sodium. This dietary pattern increases intake of potassium, calcium, magnesium, protein, and fiber and has been shown to significantly reduce both systolic and diastolic BP. Beyond BP control, evidence indicates beneficial effects on MetS, insulin sensitivity, BMI, lipid profile, inflammation, and OS [60,61,62,63].

Recent studies confirm the protective role of the DASH diet against MetS and its core components, including hypertension, hyperglycemia, central obesity, and dyslipidemia. Adherence to DASH improves glucose metabolism, reduces total and LDL cholesterol, and contributes to improved insulin sensitivity, supporting its role in MetS management [64]. Interventional studies further demonstrate that DASH is effective in reducing fatty liver indices, body weight, waist circumference, and CV risk factors, outperforming standard healthy diets in individuals with MetS [65].

Comparative evidence suggests that both DASH and Mediterranean dietary patterns reduce MetS prevalence, particularly when combined with salt restriction. While both diets improve lipid profile, glycemic control, and BP, the MedDiet may exert superior antihypertensive effects, although overall cardiometabolic benefits are comparable [66]. Meta-analytic data reinforce these findings, showing significant reductions in systolic and diastolic BP, increases in HDL-cholesterol, and decreases in LDL-cholesterol among MetS patients adhering to the DASH diet [66].

Long-term observational studies indicate that higher adherence to the DASH diet is associated with a reduced risk of ischemic stroke, an effect largely mediated by improvements in BP and body weight [67,68].

Maifeld et al. (2021) [69] evaluated the effects of a 5-day fasting intervention followed by a modified DASH diet in hypertensive patients with MetS. Compared with the DASH diet alone, fasting induced sustained reductions in systolic BP, antihypertensive medication use, and body mass index at three-month follow-up. The intervention also favorably modulated the gut microbiome, enhancing bacterial taxa and functional gene modules involved in SCFA production. Machine learning analyses identified key microbiome and immune predictors of BP response, notably *Akkermansia muciniphila* and members of the *Ruminococcaceae* family (including butyrate-producing taxa such as *Faecalibacterium prausnitzii*), alongside immune markers such as CD8^+^ effector T cells. These findings support fasting combined with a DASH-based dietary pattern as a promising non-pharmacological strategy to improve cardiometabolic health in patients with MetS [70].

This randomized study of Diao et al. (2024) [70] compared the effects of a low-calorie DASH diet and a low-calorie diet on trimethylamine N-oxide (TMAO) levels and gut microbiota in 120 obese adults over 12 weeks. Participants were assigned to a low-calorie DASH diet, a low-calorie diet, or a control group. The low-calorie DASH diet resulted in a significantly greater reduction in plasma TMAO and LPS concentrations compared with the low-calorie diet alone. Additionally, the DASH-based intervention produced a more pronounced decrease in the FBR, which was associated with changes in TMAO levels [70].

A recent study (Pourfard et al.) [71] showed that adherence to the DASH diet induces favorable alterations in gut microbiota composition, characterized by increased microbial diversity. The authors reported a significant rise in SCFA-producing bacteria, particularly *Faecalibacterium prausnitzii* and members of the *Ruminococcaceae* family, which are known for their anti-inflammatory properties. An increased abundance of *Akkermansia muciniphila* was also observed, suggesting improved intestinal barrier integrity and metabolic regulation. In parallel, the DASH diet was associated with a reduction in the *FBR*, indicative of a healthier metabolic profile. Decreased levels of potentially pro-inflammatory taxa, including *Blautia*, were reported. The enrichment of beneficial genera such as *Bifidobacterium* and *Bacteroides* was linked to enhanced dietary fiber fermentation. These microbiota changes were associated with increased butyrate production and reduced circulating levels of pro-atherogenic metabolites, including TMAO [71].

Table 3 synthesizes current evidence indicating that adherence to the DASH diet induces consistent, microbiota-mediated cardiometabolic benefits. Across studies, DASH is associated with increased microbial diversity and enrichment of short-chain fatty acid–producing taxa, including *Faecalibacterium prausnitzii*, members of the *Ruminococcaceae* family, and *Akkermansia muciniphila*, alongside reductions in dysbiosis-related markers such as the FBR, LPS, and trimethylamine N-oxide. Importantly, these microbiota changes reflect convergent biological pathways shared with other healthy dietary patterns—namely enhanced SCFA availability and attenuation of low-grade inflammation—rather than diet-specific mechanisms. The clinical relevance of DASH therefore lies in the consistency and translational impact of these shared microbiota-driven processes, which parallel meaningful improvements in BP, insulin sensitivity, central obesity, inflammatory status, and overall metabolic risk.

Although most studies report favorable effects of the DASH diet on gut microbiota composition and cardiometabolic outcomes, findings are not entirely uniform. Differences in intervention duration, study populations (obese vs. MetS vs. hypertensive individuals), microbiome sequencing approaches, and combined interventions (e.g., fasting or salt restriction) contribute to methodological heterogeneity and variable results. Short-term trials often demonstrate rapid reductions in dysbiosis-related markers such as the FBR and TMAO, whereas longer-term data on microbiota stability and resilience remain limited. Moreover, the majority of evidence is associative, and key knowledge gaps persist regarding causality, long-term microbiota adaptation, and the identification of responder versus non-responder phenotypes. Future longitudinal and mechanistic studies are needed to clarify the microbiota-mediated contribution of the DASH diet to metabolic risk reduction.

### 3.3. Microbiome and the Ketogenic Diet

Among dietary strategies for managing MetS, the ketogenic diet (KD) has attracted increasing interest due to its capacity to directly target core metabolic disturbances through profound carbohydrate restriction and induction of nutritional ketosis [72,73,74]. By shifting energy metabolism from glucose toward ketone bodies, KD improves insulin sensitivity, suppresses lipogenesis, and enhances fat oxidation—mechanisms central to the pathophysiology of MetS. Consistent reductions in postprandial glucose, circulating insulin, and insulin-to-glucagon ratio have been reported, with meta-analyses in patients with DM2 demonstrating significant improvements in fasting glucose, HbA1c, triglycerides, and HDL-c across interventions lasting up to 56 weeks [75,76].

Compared with low-fat diets, KD and very-low-calorie ketogenic diets (VLCKD) generally achieve greater reductions in body weight, triglycerides, and diastolic BP, while effects on LDL-c remain variable and context dependent. These metabolic benefits are accompanied by anti-inflammatory effects, including reductions in IL-6 and TNF-α, although concerns persist regarding long-term safety, micronutrient deficiencies, gastrointestinal symptoms, and hepatic steatosis [77,78,79,80,81,82].

Beyond systemic metabolism, accumulating evidence indicates that KD induces substantial and diet-specific alterations in gut microbiota composition and function. A recent meta-analysis by Wang et al. (2025) demonstrated that VLCKD increases gut microbial α-diversity and promotes expansion of *Akkermansia*, while concurrently reducing *Bifidobacterium*, highlighting a bidirectional and context-dependent microbiota response influenced by BMI, age, and intervention duration [83]. In contrast, a systematic review by Rew et al. (2022) consistently reported reductions in *Bifidobacterium* and butyrate-producing Firmicutes, alongside decreased fecal SCFA levels, raising concerns regarding impaired fermentative capacity and colonic health [84].

Experimental studies further support a mechanistic link between KD-induced microbiota changes and metabolic regulation. Li et al. (2024) demonstrated that KD-induced glucose intolerance in mice was microbiota dependent, as antibiotic-mediated depletion abolished dysglycemia without preventing lipid disturbances, indicating a causal role of gut microbiota in glucose homeostasis [85]. Human interventional studies corroborate these findings but reveal heterogeneous responses: while short-term KD interventions improve glycemic markers and body composition, they may also reduce microbial diversity, SCFA availability, and intestinal barrier integrity, as evidenced by increased zonulin levels and enrichment of potentially pathogenic taxa [86,87].

Notably, mechanistic studies have identified ketone bodies—particularly β-hydroxybutyrate—as direct modulators of microbial ecology, selectively inhibiting *Bifidobacterium* growth and shaping an immunologically distinct microbiota profile associated with reduced intestinal Th17 cells [88]. These effects distinguish KD from high-fat diets and underscore the role of host–microbe metabolic signaling in mediating immune and metabolic outcomes.

Collectively, available evidence indicates that KD/VLCKD exerts potent but heterogeneous effects on gut microbiota, with short-term metabolic benefits often accompanied by microbial trade-offs. Variability in intervention duration, macronutrient composition, sequencing methodologies, and population characteristics limits direct comparison across studies and constrains causal inference in humans. Key knowledge gaps include the long-term resilience of KD-induced microbiota changes, identification of responder versus non-responder phenotypes, and clarification of microbiota-driven versus host-mediated mechanisms. These uncertainties emphasize the need for longitudinal, mechanistically informed, and personalized approaches when considering KD as a therapeutic strategy for MetS [89].

Table 4 synthesizes clinical, experimental, and mechanistic evidence showing that KD and VLCKD induce pronounced but context-dependent changes in gut microbiota, with consistent short-term metabolic improvements accompanied by heterogeneous microbial responses. Across studies, ketogenic interventions are associated with weight loss and improved glycemic control, but also with recurrent reductions in beneficial taxa (e.g., *Bifidobacterium*, SCFA producers) and markers of impaired gut barrier function, particularly with longer exposure. Overall, the table highlights a trade-off between short-term metabolic efficacy and potential long-term microbiota-related risks, reinforcing the need for individualized, time-limited, and medically supervised ketogenic strategies.

Reported metabolic and gut microbiota changes following KD and VLCKD interventions are not entirely consistent across the literature. These discrepancies likely reflect substantial methodological heterogeneity, including differences in intervention duration, macronutrient composition, microbiome sequencing techniques, and participant characteristics such as obesity status, insulin resistance, or diabetes. Short-term interventions frequently demonstrate rapid microbial shifts, whereas longer studies suggest partial adaptation or resilience of the gut microbiota. Importantly, the available human evidence remains largely associative, limiting causal inference between microbiota modulation and metabolic outcomes. Key knowledge gaps include the long-term durability of KD-induced microbiota changes, the identification of responder versus non-responder phenotypes, and the relative contribution of microbial versus host-driven mechanisms, underscoring the need for longitudinal, mechanistically informed, and personalized intervention strategies.

Importantly, accumulating evidence indicates that dietary responses particularly to ketogenic and KD and VLCKD are strongly influenced by baseline gut microbiota composition, metabolic status, age, and sex, and may differ substantially between short-term and long-term interventions.

## 4. Concluding Remarks

Collectively, current evidence supports physical exercise and dietary quality as key, modifiable determinants of gut microbiota structure and function, with direct relevance to the pathophysiology of MetS. Moderate-intensity exercise and sustainable dietary patterns, particularly the Mediterranean and DASH diets, consistently promote a more diverse and functionally resilient microbiota, enriched in SCFA-producing taxa and associated with improved insulin sensitivity, lipid metabolism, BP regulation, and reduced low-grade inflammation.

Importantly, emerging data indicate that diet and exercise act synergistically, rather than independently, on the microbiota–metabolism axis, suggesting that combined lifestyle interventions may amplify cardiometabolic benefits beyond either strategy alone. In contrast, restrictive approaches such as KD and VLCKD offer rapid short-term metabolic improvements but are frequently accompanied by unfavorable microbiota shifts and impaired gut barrier integrity, raising concerns regarding long-term sustainability.

Several limitations of the current literature should be acknowledged, including the predominance of short-term interventions, substantial heterogeneity in microbiota assessment methods, and the largely associative nature of human studies, which constrains causal inference. Inter-individual variability related to baseline microbiota, metabolic status, sex, and age further contributes to heterogeneous responses.

Future research should prioritize long-term longitudinal and interventional studies, standardized microbiota endpoints, integration of multi-omics approaches, and exploration of sex- and age-specific responses. From a clinical perspective, these findings support the integration of personalized dietary strategies and structured physical activity into lifestyle counseling and public health recommendations for the prevention and management of MetS.

## Figures and Tables

**Table 1:** Key findings on the effects of physical exercise on gut microbiota and metabolic health.

Study (Year)	Population/Design	Exercise Type & Intensity	Main Microbiota Changes	Key Metabolic/Physiological Effects
Gubert et al. (2020) [22]	Narrative review	Exercise, diet, stress (environmental factors)	↑ microbial diversity; dysbiosis linked to neurodegeneration	Modulation of MGBA; effects on neurons, glia, metabolism
Hamasaki (2017) [23]	Human & animal studies	Aerobic, mind–body (Tai Chi)	↑ diversity; favorable compositional shifts	Reduced gut inflammation; vagal & HPA axis modulation
Monda et al. (2017) [24]	Review	Physical exercise (general)	↑ diversity; improved Bacteroidetes/Firmicutes ratio; ↑ SCFA-producing taxa	Improved gut barrier, immunity, metabolic homeostasis
Sohail et al. (2019) [25]	Review (62 studies)	Aerobic & resistance exercise	Exercise-responsive beneficial taxa	↓ inflammation, ↓ oxidative stress, ↑ insulin sensitivity
Bonomini-Gnutzmann et al. (2022) [26]	Systematic review (athletes)	High-intensity endurance vs. moderate aerobic	Endurance: ↑ Prevotella, ↓ diversity; Moderate: ↑ diversity	High intensity: ↑ permeability, GI discomfort; moderate exercise beneficial
Zhang et al. (2023) [27]	Review	Moderate exercise + diet	↑ diversity; ↑ beneficial metabolites	Prevention of DM2, dyslipidemia, and improved gut immune barrier
Yun et al. (2024) [28]	Interventional (4 weeks)	Moderate vs. high intensity	Moderate: ↑ *Prevotella* ↓ *Veillonella*, *Dorea*; High: ↑ *Bacteroides*, *Butyricimonas*, *Odoribacter*, *Alistipes*	↓ metabolic disease risk; intensity-dependent effects
Quiroga et al. (2020) [29]	RCT, obese children	Strength + endurance (12 weeks)	↓ Proteobacteria; ↑ *Blautia*, *Dialister*, *Roseburia*	↓ glucose; ↓ NLRP3 inflammasome; ↓ inflammation
Silva et al. (2022) [30]	Interventional (4 weeks)	Moderate vs. vigorous	Moderate: ↑ *Prevotella*; Vigorous: ↑ SCFA-producing taxa	Improved metabolic markers; exercise-specific microbial response
Allen et al. (2018) [33]	Lean vs. obese adults	Endurance training (6 weeks)	Lean: ↑ *Faecalibacterium*, ↑ SCFA; Obese: ↑ *Bacteroides*, *Collinsella*	Obesity-dependent microbiota responsiveness
Hoffman-Goetz et al. (2003, 2010) [34,35]	Experimental	Physical exercise	Not taxa-specific	↑ antioxidant enzymes; ↓ pro-inflammatory cytokines

**Table 2:** Evidence on the Mediterranean Diet (MedDiet): gut microbiota modulation and cardiometabolic effects.

Authors (Year)	Study Design/Population	Main Focus of the Study	Gut Microbiota Findings	Clinical/Metabolic Outcomes
Merra et al. (2020) [50]	Narrative review	MedDiet and human gut microbiota	↑ SCFA-producing bacteria; improved microbial balance	Anti-inflammatory and metabolic benefits
Dominguez et al. (2023) [52]	Narrative review	MedDiet in obesity prevention and management	Indirect microbiota modulation via fiber and PUFAs	↓ Obesity risk, improved metabolic health
Barber et al. (2023) [58]	Narrative review	MedDiet and gut microbiota	↑ *Faecalibacterium prausnitzii*, *Eubacterium rectale*, *Bifidobacterium*; ↓ *Blautia*	↓ Inflammation and oxidative stress; improved metabolic health
Beam et al. (2021) [59]	Narrative review	Diet–microbiota interactions	↑ *Prevotella/Bacteroides* ratio with high-fiber diets	Improved gut barrier and metabolic regulation

**Table 3:** Evidence on the DASH diet: gut microbiota modulation and cardiometabolic effects.

Authors (Year)	Study Design/Population	Main Focus of the Study	Gut Microbiota Findings	Clinical/Metabolic Outcomes
Lv et al. (2024) [63]	Narrative review/Mechanistic analysis	DASH diet in MetS and potential mechanisms	Indirect microbiota modulation via fiber and micronutrients	↓ BP, ↓ glucose, ↓ central obesity, ↓ dyslipidemia; improved insulin sensitivity
Maifeld et al. (2021) [69]	RCT; hypertensive patients with MetS	5-day fasting + DASH vs. DASH alone	↑ *Akkermansia muciniphila*; ↑ *Ruminococcaceae* (*Faecalibacterium prausnitzii*); ↑ SCFA-related gene modules	Sustained ↓ SBP, ↓ BMI, ↓ antihypertensive medication use
Diao et al. (2024) [70]	RCT; obese adults	Low-calorie DASH vs. low-calorie diet	↓ Firmicutes/Bacteroidetes ratio; ↓ LPS; ↓ TMAO	Improved gut-derived cardiometabolic markers
Pourfard et al. (2025) [71]	Observational/interventional	DASH adherence	↑ Microbial diversity; ↑ *Faecalibacterium prausnitzii*, *Ruminococcaceae*, *Akkermansia muciniphila*, *Bifidobacterium*, *Bacteroides*; ↓ *Blautia*	↑ Butyrate production; ↓ inflammation and TMAO; improved metabolic profile

**Table 4:** Effects of Ketogenic and Very-Low-Calorie Ketogenic Diets (KD/VLCKD) on Gut Microbiota and Metabolic Outcomes.

Authors (Year)	Study Design/Population	Dietary Intervention	Gut Microbiota Findings	Main Metabolic/Clinical Outcomes
Wang et al. (2025) [83]	Systematic review & meta-analysis (14 studies; obesity)	VLCKD	↑ *Akkermansia*; ↑ Firmicutes/Bacteroidetes ratio; ↓ *Bifidobacterium*	Microbiota modulation dependent on BMI, age, and duration; strongest effects in middle-aged subjects and short-term interventions
Rew et al. (2022) [84]	Systematic review (8 studies; humans)	KD	Persistent ↓ *Bifidobacterium*; ↓ butyrate-producing Firmicutes; ↓ total SCFAs (acetate, butyrate)	Potential risks for obesity, DM2, depression; impaired fermentative capacity
Li et al. (2024) [85]	Experimental animal study (mice)	Two KD formulations (KD1, KD2)	↓ SCFAs; altered bile acid profiles; KD-induced dysbiosis; glucose effects microbiota-dependent	Glucose intolerance independent of lipid accumulation; antibiotic depletion abolished glucose dysregulation
Güzey Akansel et al. (2024) [86]	Interventional clinical study (women with overweight/obesity)	6-week KD	↓ β-diversity; ↑ Firmicutes/Bacteroidetes ratio; ↓ *Bifidobacterium*, *Prevotella*; ↑ *Oscillibacter*, *Blautia*, *Akkermansia*; ↑ pathogenic genera	↓ BMI, glucose, insulin, HbA1c; ↓ fecal SCFAs; ↑ serum zonulin → impaired gut barrier
Lu et al. (2024) [87]	Clinical interventional study (T2DM patients)	KD vs. standard diet	↓ *Enterococcus faecalis*, ↓ *Escherichia coli*	↓ FPG, HbA1c, HOMA-IR, lipids; ↑ GLP-1; ↓ body weight and WC
Ang et al. (2020) [88]	Human + animal mechanistic study	KD vs. high-fat diet	↓ *Bifidobacterium* via β-hydroxybutyrate; KD-specific microbiota signature	↓ intestinal Th17 cells; immune modulation via ketone bodies
Gudan & Stachowska (2022) [89]	Narrative review	KD (animal-fat–based)	↑ *Bilophila*, *Alistipes*, *Bacteroides*; Bacteroides enterotype	Microbiota-dependent therapeutic effects in epilepsy, potential metabolic risks

## Data Availability

No new data were created or analyzed in this study. Data sharing is not applicable to this article.

## Abbreviations

BMI	body mass index
BP	blood pressure
CRP	C-reactive protein
CV	cardiovascular
CVD	cardiovascular disease
DASH	Dietary Approaches to Stop Hypertension
DM2	diabetes mellitus type 2
GLP-1	glucagon-like peptide-1
HbA1c	glycosylated hemoglobin
HDL	high-density lipoprotein
HPA	hypothalamic–pituitary–adrenal
HRR	heart rate reserve
IDF	International Diabetes Federation
IR	insulin resistance
FBR	Firmicutes-to-Bacteroidetes
KD	ketogenic diet
LDL	low-density lipoprotein
LPS	lipopolysaccharide
MetS	metabolic syndrome
MGBA	microbiota–gut–brain axis
MUFA	monounsaturated fatty acid
NCEP	National Cholesterol Education Program
NO	nitric oxide
OS	oxidative stress
PUFAs	polyunsaturated fatty acids
ROS	reactive oxygen species
SCFA	short-chain fatty acids
SNS	sympathetic nervous system
VLCKD	very-low-calorie ketogenic diet
WHO	World Health Organization
